# Unpacking complexities surrounding tobacco control policy formulation and tobacco industry interference in South Africa: a qualitative study

**DOI:** 10.1093/heapol/czaf013

**Published:** 2025-03-04

**Authors:** Mateusz Zatoński, Adam Bertscher, Allen W A Gallagher, Britta K Matthes

**Affiliations:** Department for Health, Faculty of Humanities & Social Sciences, University of Bath, Claverton Down, Bath BA2 7AY, United Kingdom; Department of Social and Policy Sciences, Faculty of Humanities & Social Sciences, University of Bath, Claverton Down, Bath BA2 7AY, United Kingdom; Department for Health, Faculty of Humanities & Social Sciences, University of Bath, Claverton Down, Bath BA2 7AY, United Kingdom; Department for Health, Faculty of Humanities & Social Sciences, University of Bath, Claverton Down, Bath BA2 7AY, United Kingdom

**Keywords:** tobacco control, tobacco industry interference, policy formulation, South Africa

## Abstract

South Africa (SA) used to be recognized as a committed leader in tobacco control (TC) having passed effective TC policies in the 1990s, but in recent years, it has been overtaken by other countries. While research suggests that the tobacco industry (TI) predominantly uses economic arguments to shape TC policy discussions in SA, TI tactics to influence policy formulation have not been examined in the peer-reviewed literature. In this study, we draw on three frameworks (health policy triangle, ‘bit in the middle’, and Policy Dystopia Model) and 20 interviews, supplemented with academic and ‘grey’ literature, to explore TC policy formulation in SA. We focus on SA’s 2018 draft TC Bill, which as of January 2025 has not been adopted. We found that despite SA’s commitment to protect TC policies from the TI’s vested interests, as set out in Article 5.3 of the World Health Organization Framework Convention on Tobacco Control, TI actors interfered throughout the policy formulation process. Participants reported efforts to shape policy alternatives by influencing the impact assessment and generating favourable evidence. To influence deliberation, they lobbied policymakers. To advocate for their preferred outcome, they sought to shape public opinion through campaigns and built alliances, for example, with the non-tobacco business community. The identified strategies were consistent with those observed elsewhere. Some were tailored to the SA context characterised by political corruption, and sensitivity around race and the legacy of Apartheid, as well as rivalry between transnational corporations and local producers. Industry actors also sought to redirect attention to TC areas (illicit trade and taxation) not led by the health sector, likely more susceptible to TI influence. The study demonstrates to policymakers, advocates, and researchers, the importance of not looking at a TC policy in isolation and of being mindful of industry efforts to exploit inherent policy-making complexities.

Key messages

- Tobacco industry actors used a wide range of strategies to influence several tobacco control policy formulation activities in South Africa, suggesting that industry interference constitutes a key barrier to progressing tobacco control.
- While strategies largely reflect those reported elsewhere, the study illustrates how industry actors adapt their approach to a country’s context, in this case, wide-spread political corruption, and sensitivity around race and the legacy of Apartheid.
- Tobacco industry actors also sought to exploit the complexities inherent in tobacco control—consisting of different policies with different processes and actors involved. Public health researchers and advocates should therefore consider policy development holistically.

## Introduction

Tobacco use continues to be a main risk factor for the onset of noncommunicable diseases, causing approximately 8 million deaths globally each year (WHO 2022). Tobacco consumption in South Africa (SA) reached a peak in the early 1990s (Tobaccotactics 2021) and although tobacco consumption has since decreased (Groenewald et al. 2007, Peer et al. 2009), 25.8% of those aged 15+ years continued to smoke in 2021 (WHO 2021a), an increase from 19.4% in 2017 (Tobacco Control Data Initiative 2023). Recently, there has been a rise in the use of newer nicotine and tobacco products, including e-cigarettes, especially among youth (Ayo-Yusuf et al. 2022).

Prior to 1993, there was minimal tobacco control (TC) legislation in SA (Malan and Leaver 2003). The Apartheid government had strong ties to SA tobacco companies, such as the SA-owned market leader Rembrandt Tobacco Company, a subsidiary of Rothmans International [which merged with British American Tobacco South Africa (BATSA) in the late 1990s; Malan and Leaver 2003]. Following the first democratic election in 1994, the new government committed to developing TC policies, seeking to tackle tobacco-related harms as a matter of racial equity, seen as important for SA’s transition to democracy (Wisdom et al. 2018).

During the early post-Apartheid transition, the government developed various TC policies and subsequent amendments—the last in 2008—making SA a TC leader on the continent at the time (Tobaccotactics 2021). It was also among the first signatories of the World Health Organization Framework Convention on Tobacco Control (WHO FCTC) (UN 2023), which includes Article 5.3 obliging parties to protect health policy from TI interference (WHO 2003). However, since 2010, SA has failed to keep pace with the implementation of recommended policies, such as ensuring 100% smoke-free spaces, sufficiently taxing tobacco products, and regulating electronic nicotine and non-nicotine delivery systems (EN&NNDS) (WHO 2023). SA has also not significantly improved its tobacco warning labels since their introduction in the mid-1990s (National Department Of Health 1994) and there is an acute lack of public sector tobacco cessation services in the country (Filby and Van Walbeek 2021).

To strengthen existing policies, the Department of Health (DoH) developed a draft Bill which was gazetted in 2018 as the Control of Tobacco Products and Electronic Delivery Systems Bill (hereafter referred to as the draft Bill; DOH 2018). Despite the draft Bill being introduced to Parliament in 2022, as of January 2025, progress has been slow and it has yet to be adopted, last being debated in September 2024 (Dhlomo 2024).

There were further proposals to increase tobacco and nicotine excise taxes (DOH 2022). Nominal tobacco and nicotine excise taxes have increased consistently since 1994. However, since 2010, they have been raised only slightly above the rate of inflation, resulting in a very small increase in real terms (Vellios et al. 2020).

SA has also signed the WHO Protocol on Illicit Trade in Tobacco Products in 2013 (WHO 2013), but has not ratified it (UN 2024). There were proposals in recent years to implement a tobacco Track and Trace System (TTS) in line with the Protocol in order to address smuggling by tracking the production, import, and export of tobacco products (Borotho 2020). However, the TTS tender process was, after repeated delays, discontinued in May 2020 (Mashego 2020, Borotho 2020).

Tobacco industry (TI) interference could help explain the lack of TC progress. Despite WHO FCTC Article 5.3, such interference remains the key barrier to TC globally (WHO 2021b, Assunta 2023). The TI has a track record of using a range of tactics and arguments to prevent, weaken, delay, and undermine public health policy (Ulucanlar et al. 2016). A media-based paper found that the TI and its front groups echo common TI arguments that SA’s draft Bill will (i) contribute to increasing illicit tobacco trade, (ii) impede access to safer alternatives for smokers, and (iii) be ineffective in reducing smoking rates (Zatoński et al. 2021). However, peer-reviewed literature on TI influence over the draft Bill is limited with the thier major tactics to influence policy-making in SA having not been researched before now.

This study aims to address this literature gap by examining TI strategies to interfere in TC policy formulation (i.e. how problems that entered the policy agenda transform into government programmes; Jann and Wegrich 2007). While we focus on the draft Bill, it cannot be considered in isolation; we thus follow Zatoński et al. (2021) in seeking to capture the complexity of TC policy formulation in SA as a whole. Using policy analysis frameworks—generally underused in the TC literature (Arabloo et al. 2018)—this study seeks to provide fresh insights into TI interference in policy formulation in a low- and middle-income country (LMIC). The policy formulation process, in general (Gilson et al. 2018), and policy analysis of TC policy-making, in particular, remain underexamined in LMICs, yet there is a growing body of research on TC policy-making in LMICs (Tam and Van Walbeek 2014, Egbe et al. 2019, Bhatta et al. 2020, Kusi-Ampofo 2021, Shahriar et al. 2023).

### Conceptual frameworks

To explore TC policy formulation in SA, we draw on three frameworks: the health policy triangle (HPT) (Walt and Gilson 1994); the ‘bit in the middle’ (BitM) (Berlan et al. 2014); and the Policy Dystopia Model (PDM) (Ulucanlar et al. 2016).

**Figure 1.**
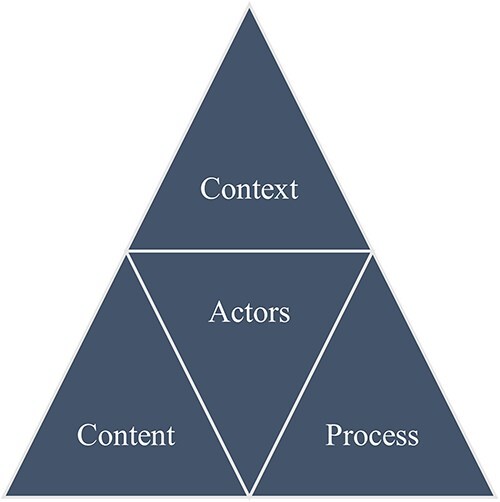
HPT adapted from Walt and Gilson (1994).

The HPT was developed to encourage health researchers to pay more attention to policy development and to assist policy analysis in LMICs (Walt and Gilson 1994). Figure 1 illustrates the HPT, which comprises four key components of policy analysis. The first component is the context of policy-making, which includes social, political, economic, cultural, and other systemic factors. The second is policy content, encompassing objectives, regulations, and similar elements. The third component is the policy process, detailing how policy-making unfolds. At the centre of the HPT is the fourth component—policy actors—who include the individuals, groups, and organizations involved in the policy-making process (Walt and Gilson 1994). The framework has been widely used in health research (O’Brien et al. 2020), including TC (Currie and Clancy 2011, Mohamed et al. 2018, Mondal et al. 2022, Tselengidis et al. 2022, 2023). While a helpful lens for structuring analysis, the HPT has been considered descriptive by nature (Moloughney and Ward 2012) and is therefore often combined with other frameworks (O’Brien et al. 2020).

The BitM framework, based on a narrative synthesis of 28 articles on health policy in LMICs, established seven activities inherent in policy formulation and adoption (Table 1) deemed ‘poorly understood, under-theorized and under-researched’ (Berlan et al. 2014, p. ii23). The framework has been used as a conceptual lens for unpacking, for example, TC policy in India (Mondal et al. 2022), alcohol policy in SA (Bertscher et al. 2018), and childhood illness policy in Kenya (Juma et al. 2015). Mondal et al. (2022) and Juma et al. (2015) combined HPT and BitM frameworks to provide a more comprehensive analysis.

**Table 1.** The policy formulation ‘bits’ in the BitM framework

Policy formulation ‘bits’	Description
Generation of policy alternatives	Generating detailed descriptions and systematic plan of action and guidelines to focus on achieving specific policy goals
Deliberation and/or consultation	Engaging with various stakeholders in conversations to explore, evaluate, and gather feedback on different policy alternatives to inform decision-making and policy development.
Advocacy for specific alternatives	Promoting and supporting particular policy choices through various efforts to influence decision makers and public opinion.
Lobbying for specific alternatives	Actively seeking to influence legislators, policymakers, or other decision makers to adopt particular policy options or proposals.
Negotiation on policy alternatives	Engaging in discussions and bargaining to reach an agreement on the details and provisions of a policy.
Drafting or enactment of policy	Creating, approving, and implementing laws, as well as allocating financial resources for the activities involved in drafting, passing, and enacting policy.
Guidance/influence on implementation	Influencing and modifying the details, implementation, and practical application of a policy after policy adoption.

The PDM was developed to bring together evidence on TI interference in policy-making (Ulucanlar et al. 2016). Drawing on two systematic literature reviews (Smith et al. 2013, Savell et al. 2014), it developed taxonomies of argument-based strategies (e.g. claiming that the policy represents a ‘nanny state’, i.e. restricting individual freedoms, or will lead to job losses) and action-based strategies (e.g. seeking access to policymakers or manufacturing industry support) that the industry employs to achieve its preferred policy outcomes. Reflecting the literature at the time, the PDM is mostly based on evidence from high-income settings. Therefore, the model was recently refined to better capture TI interference in LMICs (Matthes et al. 2021).

## Materials and methods

We used a qualitative approach to studying TC policy formulation in SA, drawing on semi-structured key informant interviews, academic and ‘grey’ literature, and using the HPT, BitM, and PDM as analytical lenses.

### Interviews: sampling and recruitment

Participants were required to be or have been involved in the policy-making processes of the draft Bill, or other relevant TC legislation, or have insights into TC policy-making or TI interference in SA. As TC policy-making is heavily contested in SA, we sought a sample that represented diverse views. Initial participants were identified through TC networks, or in the case of industry representatives, by reaching outside of TC networks to the local TI. Snowballing was then used to identify additional interviewees.

Twenty semi-structured interviews were conducted with two interviewees presenting in a single interview making a total of 21 participants. Interviews lasted between 28 and 134 min (average duration: 82 min). Participants were from researchers (R; *n* = 9); civil society (CS; *n* = 5), current or former public officials (PO; *n* = 4), TI representatives (TI; *n* = 2), and a journalist (J; *n* = 1). The TI representatives were based in SA representing transnational cooperations or the local TI. The four participants approached for follow-up comments (see below) represented four of the five groups (CS, J, PO, and R).

### Interviews: data collection

The interview schedule sought to gather participants’ experiences of TC policy formulation and TI strategies and drew on available resources on TC policy-making in SA and the PDM to capture a range of potential industry strategies. Interviewees were asked about their experiences regarding the policy formulation process of the draft Bill; the contextual factors that shaped the process; the barriers and facilitators in its development; the policy actors who were predominantly opposed, neutral, or supportive of the draft Bill; and any TI strategies to influence the draft Bill, including arguments presented to oppose it. Interviews were conducted in August and September 2019. In March 2023, four interviewees were contacted for follow-up comments and to verify our main findings. All interviews were transcribed verbatim. Ethics approval for the study was obtained from the Research Ethics Approval Committee for Health (REACH) at University of Bath (Reference: EP 18/19 012).

### Other sources: data collection

We searched online resources for any TC developments between 2010 and 2022, including websites of the government (gov.za) and the Parliamentary Monitoring Group (pmg.org.za), and media outlets (e.g. The Mail & Guardian, TimesLIVE, Daily Maverik, and News24). We also searched for relevant academic literature.

### Data analysis

We coded interview transcripts to the key components of the HPT (Walt and Gilson 1994): (i) context of policy-making, (ii) policy content, (iii) policy process, and (iv) policy actors. To avoid repetition, we did not include a section on actors in the results, instead mentioning relevant actors throughout the findings.

We also coded the data to the BitM framework (Berlan et al. 2014). We used Berlan’s seven activities (Table 1) as a starting point, adapting them as part of the analysis to best capture TC policy formulation in SA. We omitted the last three steps as there was insufficient evidence (5—Negotiation on policy alternatives, 6—Drafting or enactment of policy, 7—Guidance/influence on implementation), likely due to the limited progress of the draft Bill. We further combined two steps (2—Deliberation and/or consultation and 4—Lobbying for specific alternatives) to avoid repetition.

Lastly, we coded the transcripts to the argument- and action-based strategies of the PDM. We used the latest version (Matthes et al. 2021) as a starting point while allowing for inclusion of additional strategies or nuances not captured previously.

The interview data were triangulated with other data, namely peer-reviewed articles, books, media articles and government documents, where possible. When claims remained unverified through additional evidence, we acknowledge this. NVivo v12 (QSR International) facilitated data analysis.

## Results

In the following, findings are presented according to three key components of the HPT (context, content, and process). The process section is structured according to the adapted BitM framework. The final section is on the identified action-based and argument-based strategies applying the PDM.

### TC policy-making context: social, economic, and political factors

The ‘legacy of Apartheid’ left SA with high inequality, with the country’s wealth concentrated in the minority white populations, although modest gains have been made since (Squazzin 2021). To redress the economic exclusion of SA’s black population during Apartheid, since 1994, the government has implemented policies of black economic empowerment (BEE) as a form of affirmative action to encourage participation in the economy. As such, economic arguments, often raised by TI actors (Box 1), are sensitive matters (CS1, J, R6/9).

**Box 1. Background information about the main TI actors in SA**

Three of the largest transnational tobacco companies—Philip Morris International (PMI), BAT, Japan Tobacco International (JTI)—are present in SA. BATSA has been dominating the market as the leading manufacturer and distributor for decades; however, recently its market share has slightly dropped. In 2022, BATSA held 61.2% of the cigarette market, followed by JTI with 11% and PMI with 8.9% (Euromonitor International 2023). In addition, there are several smaller, local tobacco companies that entered the market in the last 15 years, offering cheaper brands.

There are organizations representing different sectors of the TI in SA: 1. the Tobacco Institute of South Africa (TISA), founded in the 1990s and defunct in January 2020, representing the tobacco supply chain, including transnational tobacco companies, and 2. the Fair-Trade Independent Tobacco Association (FITA), founded in 2012, representing small local tobacco companies and farmers. There appears to be rivalry between them, with each blaming the other for the high level of illicit trade in the country (R3, TI1/2). While some participants suggested it was mostly FITA members (R6/9, TI2), there were also suspicions that both were involved (PO2, R3/6, TI1).

TISA was generally seen as representing ‘white’ foreign corporate interests, whereas FITA is perceived as representing small local ‘black’-owned tobacco farmers and producers (CS4, J, R5/7, TI1). This suggests a racial dynamic intersecting with powerful transnational corporations dominating local producers. More recently, the transnational companies sought to counteract this impression (J): when TISA was disbanded, the South Africa Tobacco Transformation Alliance (SATTA) was founded with aims including ‘support[ing] the local leaf industry’ and ‘supporting the transformation of rural communities’ (SATTA n.d.). BATSA and the Black Tobacco Farmers’ Association are among its four members (SATTA n.d.).

At the same time, illicit tobacco trade has increased from <5% in 2009 to >30% in 2017, then peaked 60% in 2021, and slightly decreased in 2022 (Van Walbeek 2020, Filby and Van Walbeek 2022, Vellios 2022, Vellios and Van Walbeek 2024) - a development that features strongly in TC discussions. Researchers suggest that the increase cannot be attributed to tax increases as the inflation-adjusted excise tax was stagnant (Van Walbeek 2020, Vellios et al. 2020, Filby and Van Walbeek 2022). The responsibility for illicit trade has been highly contested, with smaller local companies (represented by the FITA) and transnational corporations [at the time, represented by the Tobacco Institute of South Africa (TISA) which is now defunct] blaming each other (Box 1). There have been significant tensions surrounding this topic with alleged death threats and assassination attempts on managers in companies within FITA (CS4, TI1, Jordaan 2019, Wiener 2019), which, according to an interviewee, also raised concerns about researchers’ safety (R6). The situation was further complicated during the COVID-19 pandemic when the government banned tobacco sales for 5 months, which was seen by researchers as contributing to the above-mentioned increase in illicit trade (J, PO2, R6, Filby and Van Walbeek 2022).

TC policy-making also needs to be seen against a backdrop of widespread political corruption and fraud—referred to as ‘State Capture’—in SA’s government, public agencies and state-owned enterprises during former President Zuma’s administrations (2009–2018) (CS2/4/5, J, PO2, R1/2/3/5/6/7/9, TI1/2, Rowell et al. 2022, Van Wyk 2022). A major political incident is described in Johann van Loggerenberg’s book, *Tobacco Wars* (Van Loggerenberg 2019): a civil servant from the State Security Agency was recruited by BATSA to spy on FITA, while at the same time having an intimate relationship with van Loggerenberg, then a senior official in the South African Revenue Service (SARS) investigating the illicit trade in tobacco (CS1/2/4/5, PO2, R1/2/3/5/6/8).

Furthermore, TISA and BATSA reportedly employed a private firm to spy and conduct surveillance on and sabotage FITA members (Gomis and Rowell 2021, Snyckers 2021). The police were used to conduct raids and delay FITA distribution trucks while escorting BATSA’s trucks (CS2, PO2, R5, TI1, Chapman et al. 2021, Rowell et al. 2022). BATSA was also found to have engaged in tax evasion (R9, TI1, Rowell et al. 2022).

Close links between political leaders and TI during the period of State Capture under Zuma’s administrations are also relevant for understanding TC policy-making in SA. The closeness between TI and political leaders is perhaps best illustrated by the son of former President Zuma having served as director of a FITA-member tobacco company (R2/3, TI1, Rees 2011, Khoza 2014). Several participants also suspected that the TI made political donations (CS1/4, PO2, R3/5) and an industry representative confirmed that tobacco companies’ managers donate to political parties in ‘their personal capacities’ (TI1). Media reports revealed that a director of a FITA member accused of being involved in illicit trade (Dolly and Cowan 2018, Pauw 2018), donated money to the Economic Freedom Fighters and ANC, allegedly giving him access to political elites (TI1, Haysom 2019). Yet, SA law does not require disclosures of political donations, and so they largely remain unknown (CS1/4, R3)(The Presidency 2019).

### Content: the draft Bill and other TC policies

The draft Bill was developed by the DoH to align with the implementation of recommended policies and TC standards and to tackle the rise of EN&NNDS (PO1/3). It contains the following key measures (DOH 2022):

– 100% smoke-free indoor public places and certain outdoor areas,
– ban of sale of cigarettes through vending machines,
– standardized packaging with graphic health warnings and pictorials,
– ban on display at point-of-sale, and
– regulation of EN&NNDS.

The other two TC areas often discussed in relation to the draft Bill are taxation and illicit trade. Taxation falls under the remit of the Treasury, which determines, for example, tobacco excise tax in the annual budget speech (CS5, PO2), and there are two separate draft tobacco-related bills: one regarding tobacco products and the other on nicotine taxation (National Treasury 2022). The lack of substantive tax increases in recent years has led researchers to call for higher rates (Filby and Van Walbeek 2022, REEP 2022, Van Walbeek 2022).

In tackling the illicit tobacco trade, SARS plays a crucial role in SA. Although formally not a policy-making body, it has powers to create, advertise, and award tenders, (CS4, R5) such as in the case of the tobacco TTS. A TTS involves internationally recognized standards to monitor cigarette production and sales by marking them with a unique, secure, and non-removable code or stamp, as required by the WHO Protocol to Eliminate Illicit Trade in Tobacco Products (WHO 2013). In SA, a TTS would replace the current ‘diamond stamp’ system described as ‘honesty-based’ and ‘ineffective’, since factories self-declare volumes of cigarettes produced which are then taxed (R5/9, TI2, Finance Standing Committee 2019).

### Process: industry interference in policy formulation

A reform of the Tobacco Products Control Act of 1993, most recently amended in 2008, has been discussed for over 10 years (CS5), but consecutive governments have, since 2010, instead prioritized the National Health Insurance policy (CS1/2/3/4, PO1/2/3, R2/3/7). The DoH responsible for the draft Bill has generally lacked resources and capacity (CS1/2/3/4/5, PO2/4, R2/8), and in the case of TC, ‘[t]here is no dedicated budget, there is no dedicated staff’ (PO2).

#### Generation of policy alternatives

In 2016, the Cabinet requested the DoH to conduct the Social Economic Impact Assessment (SEIA) on its proposed draft Bill (PO3, R8). The SEIA system, which had replaced the regulatory impact assessment in the same year (PO1, DPME n.d.), aimed to ensure that future policies aligned with the long-term 2030 National Development Plan (PO1). The SEIA involves a cost–benefit analysis, emphasizing social and economic implications of the proposed policy. The DoH was responsible for conducting the SEIA and received support and guidance from the Department of Planning, Monitoring and Evaluation (DPME), located within the Presidency (PO3).

The DoH requested support from researchers at the University of Cape Town (UCT) for part of the SEIA (PO1/3, R8) and it consulted eight departments within the Forum of South African Directors-General to encourage policy integration across government. The DPME sought input from medical and public health experts (PO1) and from TI (PO1/2/3, R6/7/8) before the SEIA was published in March 2018 (DPME 2018).

On the last day of the data collection for the SEIA, the TI inundated the DoH and researchers with a vast number of documents and comments (PO2, R6/7/8). Some of the evidence cited by the TI was perceived to be poor quality or inappropriate, for example, referencing studies that did not substantiate claims made (R7). A researcher involved in analysing the input suggested that some appeared to have been copied directly from tobacco company submissions in other countries (R7).

The TI also attempted to discredit the SEIA (PO2/3, R2/7). Industry actors complained about the process, claiming that it lacked formalization and proper consultation from industry demanding, for example, that every single tobacco company be approached directly (PO3). They also criticized the SEIA findings and draft Bill, claiming that the draft Bill went against SA’s 2030 National Development Plan, which prioritizes BEE (CS3/4, J, R3/7), as some tobacco companies were at least 50% black-owned and -managed (R7). Those who represented the TI reported that they also contacted and met with researchers involved in the SEIA (TI2). Other interviewees recalled that the TI criticized them in the media (PO2), wrote them letters (R7), and complained to their employers (R8). One participant claimed that the Presidency was approached by the TI to discourage the SEIA from occurring (PO2). Participants described these efforts as ‘delay tactics’ (PO3, R2/8).

The main TI argument was that the Bill would not decrease tobacco consumption if illicit products remained easily accessible. Hence, instead of a new law, better enforcement of existing policies would be needed (TI1/2). The TI’s preferred approach was to avoid new regulations while furthering partnerships with government regarding enforcement and illicit tobacco trade. This is reflected in the wide range of arguments the TI and linked groups used (see PDM section), not only covering the content of the draft Bill, but also illicit trade. A participant noted ‘[t]his was their tactic to refocus government officials; take them away from the bill, and rather project their interests towards illicit trade’ (PO2).

The TI also leveraged arguments that the TC policy was ‘coming from a position of privilege’ (R7) and would be imposed from a Global North perspective: while it could work in well-developed urban areas of SA, the same standards should not be applied to less developed areas (CS5, R7, TI1/2). Participants suggested that to underpin TI arguments, the transnational companies and TISA sought to generate favourable evidence, mostly linked to the illicit tobacco trade, generally exaggerating its extent (CS4, TI1, R1/6/8). Participants mentioned four TI-funded pieces of research (Table 2) and a fifth one on illicit trade and sales ban (Payi 2022) with suspected industry funding (R6).

**Table 2.** Four TI-funded pieces of research

Market research company	Funder	Focus	Public availability	Key claims of reports	Criticised by researchers?	Sources
Econex (now part of FTI Consulting)	TISA	TI contribution to job	No, but referenced in illicit trade reports	Unclear	n/a	R7, TISA 2018a, Consultancy.Co.Za 2019
IPSOS	TISA	Cost of illicit tobacco trade in SA	No, but presented	Unclear	Yes	CS2, J, PO2, R1/2/5/6/8/9, T1/2, TISA 2018b
Econometrix	BATSA	Tobacco exercise tax	Unclear	Increasing tobacco excise tax could increase illicit trade, meaning that the governments would lose billions in tax revenue	Yes, claims are contrary to researcher from the University of Cape Town (UCT)	R7, Van Walbeek 2020
Victory Research	JTI	Standardized packaging	Unclear	There is low public support for tobacco-standardized packaging in SA	Yes, research methods flawed, report partly identically to JTI-commissioned research by a UK-based firm	R6/7, Vellios and Filby 2019, Victory Research & JTI 2019, JTI 2018

While study reports were generally not made public, key claims were widely disseminated (CS4, J, R7). According to interviewees, the media—generally seen as business-friendly in SA—played a crucial role in this as journalists would tend to report industry figures without critically analysing them and omit a public health perspective (CS1/4, R2). Participants suggested that journalists could have been incentivized, through payments or gifts, to write TI-friendly stories (CS1/4, J, R2). Importantly, there were some media reports by journalists, opinion pieces and a ‘letter to the editor’ written by public health advocates, which criticized the TI and supported the draft Bill (Modjadji and Cullinan 2018, Kalideen 2018a, 2018b, Van Dyk 2018a, 2018b).

In addition to efforts to influence the draft Bill, participants suggested the TI also sought to influence SARS regarding the illicit tobacco trade (CS2/4, PO2). They reported that the government was encouraged to implement systems similar to Codentify (PO2), the industry’s own TTS system that has been found to be ineffective and inconsistent with the WHO Protocol to Eliminate Illicit Trade in Tobacco Products (Joossens and Gilmore 2014). There were also suspicions that the TI sought to convince SARS to turn the tender into a public–private partnership, which would allow industry to partner with government (R9).

#### Deliberation and/or consultation and lobbying

Throughout the process, the TI sought to meet and build relationships with various policymakers and POs (CS4, J, R2, TI1/2). In the words of a TI representative,

I will knock on parliamentarians’ door, I will set up meetings with them in parliament, I will go to government departments, to their offices, I will sit with them, I will lobby them, I will talk to them about tobacco. (TI2)

They further explained that they had approached the Treasury, the Department of Trade and Industry, and the Department of Agriculture to ‘talk about the Bill’ (TI2), although these departments were not formally involved in its development. Other interviewees found the link to the Treasury particularly concerning; a researcher reported ‘[t]hey are playing the Treasury. [For] every information, Treasury has a reference to [TI] data’ (R2). TI representatives were also reported to have ingratiated themselves with parliamentarians with participants recalling that, during a meeting in parliament, a TI representative gave a parliamentarian a cake for their birthday (CS2, R7) and PMI invited parliamentarians ‘to Switzerland to see their [tobacco] plant’ (CS3). The approach to the DoH was markedly different: there were repeated threats of legal action, arguing, for example, that standardized packaging would be unconstitutional (R4/7, PO2/4). A former public health official recalled that ‘[e]very single thing we’ve done has been legally threatened’ (PO2).

After the SEIA was completed, the draft Bill was gazetted in May 2018 and stakeholders were given three months to submit comments. The TI made multiple submissions (TI2, CS5). Participants reported there were several duplicate submissions (J1) and suggested that the industry may have encouraged individuals or organizations to submit comments (CS3/5). It was also noted that concerns about the draft Bill’s negative impacts on sectors such as hospitality and tourism, repeatedly mentioned by the TI, were, in fact, not raised by organizations from these sectors (R1).

While there was no public hearing by parliament on the draft Bill at this stage, there were public hearings on nicotine tax for EN&NNDS during which industry representatives would generally argue that higher taxes would lead to increased illicit trade and decreased government revenue (TI2, R5/6/8). It was noted that, when they engaged in meetings with policymakers or officials, industry organizations would bring several representatives—‘they will have four people, and we were two and that’s our whole organization’ (CS4). This might have given the impression of greater support for the TI position than was the case. Moreover, a participant reported that industry recommendations for excise tax lacked supporting evidence (R2).

Some government departments and agencies, notably the Treasury and SARS, were seen as being more open to engaging with the TI, likely facilitated by other regular interactions (CS2). For example, the TI attends quarterly meetings with SARS—called ‘Tobacco Industry Forum’ (CS2, R5, TI1/2)—with FITA and (later) TISA, which are, according to a TI representative, ‘purely on taxation issues, illicit trade issues, and things like that’ (TI2).

#### Advocacy for specific alternatives

When the draft Bill was gazetted in May 2018 and stakeholders were given three months to comment, in addition to submitting responses, as mentioned earlier), the TI also started several initiatives seeking to shape public opinion on the draft Bill.

First, JTI launched the #HandsOffMyChoices campaign (CS1, R5, TI1) which argued that (I) standardized packaging will increase illicit trade and decrease revenue for legitimate businesses, (II) there will be risks of imprisonment for smoking in banned spaces, (III) there is ‘little actual evidence’ of EN&NNDS’ harm, and (IV) the display ban will not work and will negatively impact small businesses (JTI n.d). Signatures for a petition were collected and submitted in August 2018 JJTI n.d. The campaign garnered considerable media attention, sparking discussions about TC and excise tax (CS1, R5/6, Zatoński et al. 2021).

Secondly, coinciding with the TISA-funded IPSOS study (Table 2), TISA launched the #TakeBackTheTax campaign in July 2018, suggesting that instead of the draft Bill, the government should prioritize tackling illicit tobacco trade (R1/5/6/8, J, TI2). This campaign was widely disseminated through billboards and social media. It was led by Yusuf Abramjee, a self-proclaimed ‘anti-crime activist’ (CS4, J, R5/7; Tobaccotactics 2020b, Zatoński et al. 2021).

In addition, a TISA ally, the Food and Allied Workers Union (FAWU), launched the #NotJustAJob campaign, claiming that the draft Bill would lead to job losses, particularly in the agricultural sector (CS1/4, R9, Zatoński et al. 2021).

More recently, in response to the draft Bill’s introduction in parliament in 2022, Limpopo Tobacco Processors (a member of SATTA, the ‘new’ TISA—Box 1) launched the StopTobaccoBill campaign, including a petition. The campaign claims that the draft Bill would criminalize smokers for smoking in the ‘wrong place’ (Limpopo Tobacco Processors 2023), an argument made previously by JTI’s #HandsOffMyChoices campaign.

Participants suspected that the TI hired public relation firms to advise them on these campaigns, although none were named (CS1/4, TI2). A TI representative openly spoke about working with ‘media consultants’ (TI2).

During policy formulation, the TI also attempted to fund research and scientific events to create an industry-favourable environment. The Foundation for a Smoke-Free World (FSFW) (rebranded to Global Action to End Smoking in May 2024) (Tobaccotactics 2024b), which was solely funded by PMI (Legg et al. 2019), was set to donate over R1 million to the UCT’s Department of Psychiatry and Mental Health to establish the African Centre of Excellence for Smoking and Mental Health in 2018 (J, R1/3/6, Van Dyk 2019). This attempt was thwarted by academics at the institution who objected, although some of the money was spent (R3). Since then, in 2019, the UCT has implemented a policy prohibiting university staff and affiliates from accepting any research funding from the TI (Reep 2020). Also, in 2018, the FSFW awarded a R1.2 million grant to the University of Stellenbosch’s Business School focused on ‘research and projects regarding quitting or switching’ (Foundation for a Smoke-Free World 2022). In 2021, the FSFW awarded funding to The Foundation for Professional Development, which in turn sponsored SA’s 7th Tuberculosis Conference (Van Dyk 2022). After this became public, the organization stated they would pay back unspent funding and withdraw its research from journals (Van Dyk 2022).

The TI targeted academics, often in business schools. For example, PMI sought to share their latest research findings with at least two academics (CS1/3). A TI representative shared that they regularly engaged with the UCT’s Business School to talk about tobacco trade (TI1). In one instance, PMI was listed to cohost a seminar with UCT’s Graduate School of Business (R3). After an academic complained about this to the School’s Dean, the event was cancelled (R3).

The TI sought to foster connections with business organizations. A TI representative explained ‘I’ve done some stuff with Business Leadership South Africa, an organization of the top 200 businesses on the stock exchange.’ (TI1). They also reported seeking to build partnerships with business associations, such as the Chamber of Commerce, Business Unity South Africa, and the business-friendly think tank Free Market Foundation, to ensure businesses speak with ‘one voice’ (TI2).

The TI engaged with SA’s largest agricultural organization, AgriSA, the FAWU (which had run the above-mentioned #NotJustAJob campaign), and the South African Informal Trader’s Association (TI2). There are also suspicions that they garnered support from a taverners association, which criticized the draft Bill (Magubane 2018). A participant reported, ‘they get [taverners and trader’s associations] together and wind them up. [Then] they think all their jobs are at stake and they submit a lot of comments’ (CS5).

To enhance its image with policymakers and the public, the TI also engaged in corporate social responsibility (CSR) activities, often linking this to black farmers. The TI representative mentioned conducting ‘new development projects of especially black farmers… where they get trained on tobacco farming but also on maize farming, on vegetable farming and so on’ (TI2). A participant from civil society suggested that BATSA would support the Black Tobacco Farmers’ Association to ‘secure land to farm tobacco leaf’ (CS3). Participants recalled activities linked to illicit trade, including gifting sniffer dogs and scanners and providing training for SARS staff (R5/9).

### Applying the Policy Dystopia Model

Participants’ accounts suggest that TI actors used ‘argument-based strategies’ reported elsewhere (Table 3): they evoked economic/development arguments, such as that the point-of-sale advertising ban would lead to job losses, and illicit trade-related arguments, suggesting that standardized packaging and tax raises would increase illicit tobacco trade. They argued that banning point-of-sale advertising criminalizes informal street vendors and that standardized packaging violates the intellectual property of brands, is unconstitutional, and blocks the right to information. TI actors also used politics and governance narratives, e.g. the draft Bill represents ‘nanny state’ policies, and social justice narratives, including that increasing tobacco tax will negatively impact black farmers. They also stated that TC penalizes an industry that contributes to the economy and creates jobs.

**Table 3.** Argument-based strategies based on the PDM framework

Discursive strategy	Domain	Arguments from PDM	Arguments identified*	Example illustrating arguments
Expanded/created				
Unanticipated costs to economy and society	The economy and development	The policy will lead to lost sales/jobs.	Point-of-sale advertising ban will hurt the job market (CS4/5, J, R5)	‘Industry clearly goes out to [business associations] and all those other groups and say, “your livelihood is going to be destroyed”. And then they tell lies, for example like if you ban smoking, you know there’s 300 000 jobs at stake.’ (CS5)
		The policy will lead to lost or unreliable tax revenues.		
		The policy will damage the country’s economy/development.		
		The policy will worsen the situation of farmers.		
	Law enforcement/tobacco smuggling	The policy will increase illicit trade.	Standardized packaging will further drive illicit and counterfeit cigarettes (CS2, R5).	‘… the industry’s strength, and it’s typical of everywhere you go, is illicit trade. So plain packaging will increase illicit trade.’ (CS2)
			Tax increases will increase illicit tobacco trade (R2/5/6/8, TI2).	‘[The tobacco industry says] Freeze tax at where it is or decrease taxes and then you will solve the illicit trade problem.’ (R2)
			To solve illicit trade, freeze or decrease tobacco taxes which will increase government revenue (R2, TI2).	‘I presented to parliament and I asked them for […] a tax freeze for three years on not increasing excise. And I explained to them why. I gave them the history of tax increases on tobacco, the growth of illicit trade, the link between high prices and illicit trade and the bad enforcement. […] And I asked them for all of this to put a tax freeze because you’re actually going to get more money if you don’t increase taxes. And it will allow the legal industry to get the volumes back rather than shedding it to the illegal industry who don’t pay tax.’ (TI2)
		The policy will criminalize the public.	Banning point-of-sale advertising criminalizes informal street vendors who rely on the tobacco trade for their livelihoods (CS5, TI1/2).	‘They [the media] buy the lie of the industry saying […] you’re going to criminalize … oh the poor trader, he’s got children to feed and you’re going to criminalize him.’ (CS5) ‘The display ban will criminalize thousands of small businesses.’ (TI2)
	The law	The policy breaks intellectual property laws.	Standardized packaging violates the intellectual property of brands (TI2).	‘If you’ve got plain packaging, we believe that you encroach on the property of the rent owner…, those packets don’t belong to the government.’ (TI2)
		The policy breaches trade agreement(s).		
		A public body is acting beyond its jurisdiction.	Standardized packaging is unconstitutional (R2).	‘Lawyers [from law firms] that keep the patents [for the tobacco industry] write an opinion piece on what they think about plain pack and trademark in South Africa, they … [argue that] the constitution will guarantee your trademark and it’s a national law….’ (R2)
			Standardized packaging blocks the right to access information (CS3).	‘[Tobacco companies] are talking about packaging. They are raising the issue of the right to information. To giving information. They are quoting this act and that act and so on.’ (CS3)
	Politics and governance	The government is anti-free enterprise.		
		Nanny state/slippery slope.	The draft Bill represents policies of a nanny state (CS5, J, R3).	‘The other phrase that is used by the industry and so the media buys it. “Nanny state. We want to regulate anything. We’re becoming a nanny state.” They use it for other legislation as well, but certainly for tobacco control.’ (CS5)
		The government is unreasonable/unaccountable.		
		The policy is not in the national interest.		
	Social justice	The policy is unfair for smokers.		
		The policy is regressive.	Increasing tobacco tax will negatively impact black farmers (R5).	‘[Tobacco tax] is bad [for] black tobacco farmers.’ (R5)
Unintended benefits for undeserving groups		Smugglers will profit from the policy.		
		Big business will profit from the policy.		
Unintended costs to public health		The policy will be counterproductive.		
Penalization of a reputable/ legitimate industry		This policy penalizes a reputable and legitimate industry that creates jobs, invests in the country, is a crucial taxpayer, helps farmers, etc.	TC policies penalize a legal industry that is contributing to the economy (PO2). Tobacco provides economic opportunities, which creates jobs (CS1, R2/3/9).	[Industry claims that] ‘It’s a legal entity’, ‘They’re bringing money to the economy.’ (PO2) ‘[Industry claims that] tobacco would give economic progress, there is no employment, embrace tobacco and you will get jobs.’ (R2)
Contained/denied				
Intended public health benefits		There is not (good) enough evidence. (LMIC-specific reason: the evidence comes from the Global North)	Standardized packaging did not reduce consumption in other countries, such as Australia (TI2). TTS did not work in other countries, such as Kenya (R5). There is a lack of evidence that EN&NNDS are harmful (JTI n.d.).	‘In Australia where [standardized packaging] has been the longest, it is not having an impact on consumption….’ (TI2) ‘It was a BAT press release on track and trace [TTS] where they basically said … Kenya did the same thing and it didn’t work.’ (R5) ‘One of the most significant changes to the existing tobacco legislation is the inclusion of vaporizers and e-cigarettes as tobacco dispense devices, which means they will be subject to the same extreme restrictions as regular tobacco products. This is a problem as there is little actual evidence to back up the idea that these devices are as dangerous as regular cigarettes.’ (JTI n.d.)
		The policy will not work. (LMIC-specific reasons: it might work in a developed but not in a developing country; low state capacity)	Standardized packaging works in high-income countries, but not in SA due to how the informal economy operates, such as with street vendors (TI1). Restricting point-of-sale advertising is unenforceable in the informal economy (e.g. with street vendors), which is contrasted to the formal economy (e.g. with retailers) that operates in developed urban areas similar to the formal economy in other developed countries (TI1/2). TTS will not work in SA because it is technically behind (TI1/2). TTS will not work because of informal traders (TI1/2).	‘you’ve got to understand, South Africa is not like Europe … in the informal space. […] Hawkers, street vendors and all of that, they really don’t have display units, it’s all on a table so know, how do they then hide what they’re selling?’ (TI1) ‘Now, let’s get realistic about that in the South African context where 80% of all cigarettes in South Africa are sold in the informal sector … [which] consists of small shops, tabletops, shabeens, street vendors, roving vendors at taxi ranks […] … where does he go with his cigarettes … if he can’t display it? So, it is just a nonsensical thing to do. We mustn’t think about the first world part of our country where you go into the mall across the shop, where these shops are, can probably put the cigarettes behind the counter or hide it in the cupboard below. Or at the back or whatever. That’s not the reality in this country. So, if you have a display ban in this country … how are you going to enforce it?’ (TI1) ‘It’s going to be expected of retailers, small businesses, to implement a [track and trace] system, they don’t have the technology, they sell their cigarettes in single sticks. Track and trace can’t trace single sticks, it’s not possible.’ (TI2) ‘You can’t introduce this [TTS] system in South Africa, we have a completely different business dynamic to Europe where it’s been implemented in that, we have a lot of informal trade.’ (TI1)
		The policy is not needed.	Instead of TC policy, government should create education to effectively tackle tobacco consumption (TI2).	‘… far too little emphasis placed on education. I, for example, try to partner with our education department. Teach them from a young age about the harmful effects of stuff that they do, of which smoking is one. And I think that, that’s a far better investment to make….’ (TI2)
			There is a lack of capacity for enforcement of designated smoking area laws or point-of-sale advertising in SA (R8, TI2).	‘I think it’s [tobacco control is] badly enforced […] I think public place smoking is one where you can have better enforcement […]. There are many places at the point of sale where the law is quite clear on what can and cannot happen at the point of sale. At some places it’s well enforced but maybe at some places there can be better enforcement of point-of-sale advertising.’ (TI2)
Expected TI costs (not mentioned by the industry)		The policy will lead to reduced sales.	Not mentioned	
		The cost of compliance will be high.	Not mentioned	

* All arguments were identified by participants except for one, which we found on JTI’s #HandsOffMyChoice website, that claims there is a lack of evidence that EN&NNDS are harmful.

TI actors further argued that TC will not have the intended public health benefits, suggesting that standardized packaging did not reduce consumption elsewhere and TTS did not work in other countries. They also mentioned that standardized packaging works in high-income countries, but not in SA due to the informal economy, and that TTS will not work because of SA’s lack of relevant technology and a high number of informal traders. Lastly, they stated that the draft Bill was not needed because government should instead improve enforcement and invest in education to effectively tackle tobacco consumption.

Participant data indicate that TI actors used most of the ‘action-based strategies’ previously reported elsewhere (Table 4): they worked with and through industry bodies (most notably, FITA and TISA) and other organizations seeking to build an industry-supportive coalition while undertaking efforts to undermine the public health coalition, for example, by intimidating researchers. They produced and widely disseminated industry-friendly information, notably by funding research and running campaigns to influence public opinion, while challenging public health evidence and undermining the research activities of their opponents. They sought to build and maintain connections with policymakers and used opportunities to formally contribute to policy-making, including by submitting comments. In addition, they sought to ensure a positive image, by working with consultants, engaging in CSR projects (particularly targeting black farmers), and offering support to the government in tackling illicit trade despite suspicions that industry actors were involved in or facilitating illicit trade. At the same time, the industry sought to undermine their opponents’ reputations, for example, criticizing individuals in the media. Legal action was the only action-based strategy listed in the PDM which was not identified here, possibly because no policy has been adopted, although the DoH was threatened with legal action.

**Table 4.** Action-based strategy based on the PDM framework

Action-based strategy		Examples mentioned by participants
Coalition management	Building an industry-supporting coalition (constituency recruitment and fabrication)	– Working through TI organizations (TISA, FITA, SATTA)
		– Working with other groups like the FAWU, South African Informal Traders Association, and AgriSA (the largest agricultural organization) (CS1/4, TI2, R9). Suspicions that they encouraged other groups, such as informal traders, to contribute to SEIA (CS5)
		– Building links with business schools and connections with business organizations (TI1/2)
		– Efforts of the FFSW to work with universities and researchers and fund a conference (J, R1/3/6)
	Managing the public health coalition	– Complaining to employers of researchers involved in SEIA (R8)
Information management	Producing a skewed evidence base/intelligence gathering	– Four TI-funded pieces of research and a fifth one with suspected industry funding (Table 3) (CS4, J, PO2, R1/2/5/6/7/8/9, TI1/2)
	Amplifying industry-sponsored information and misleading/confounding information	– Wide dissemination of key claims of industry-funded research (CS4, J, R7) – Running and wide dissemination of campaigns (e.g. #HandsOffMyChoices and #TakeBackTheTax) sharing misleading industry claims (CS1, J, TI2, R1/5/6/8) – Industry submissions to SEIA considered inappropriate and of poor quality (R1/2/7)
	Contesting public health evidence/efforts to silence opponents	– Contesting evidence on EN&NNDS, effectiveness of display bans, etc.
		– Approaching the Presidency to stop research from occurring (PO2)
		– Attempting to stop journalists from writing unfavourably about tobacco issues (J)
	Credibility—concealing links with information/evidence	– The industry might have paid individuals to submit to SEIA (CS3)
	Managing longer-term media relations	– Journalists might have been incentivized through payments or gifts to write industry-friendly stories (CS1/4, J, R2)
Direct involvement and influence in policy	Gaining/maintaining access to policymakers	– Building relationships with policymakers, ‘knocking on doors’ of parliamentarians and ministries (TI2)
		– Giving a birthday cake to a parliamentarian (CS2, R7)
	Using incentives and pressures	– Threatening DoH with legal action (R4/7, PO2/4)
		– Inviting parliamentarians to their business offices in Switzerland (CS3)
	Engaging as an actor in government-decision-making/legislative process	– Submitting contributions to SEIA (CS2/5, PO2, R6/7/8, TI1)
		– Contributing to public hearings on tax (R6/8/9, TI2)
Litigation		- n/a
Illicit trade		– Suspicions of involvement in illicit trade (TI1/2, R3/6/9)
Reputation and image management	Building, maintaining, and rehabilitating industry reputation/image	– TI might have worked with PR firms (CS1/4, TI2), TI representative stated working with ‘media consultants’ (TI2)
		– Engaging in CSR activities, especially around black farmers (TI2), also offering support to the Black Tobacco Farmers’ Association (CS3)
		– Gifting sniffer dogs and scanners, providing training for SARS staff (R5/9)
	Discrediting public health organizations and advocates	– Discrediting researchers involved in SEIA (PO3, R2/7), including name-calling in the media (PO2)

## Discussion

Drawing on interview data and academic and ‘grey’ literature, this article explored TI efforts to influence SA’s draft TC Bill, which was gazetted in 2018 but has not been yet been adopted (February 2025). We found that TI actors sought to shape policy alternatives, for example, using favourable evidence they generated and influencing deliberation by lobbying policymakers and officials. To advocate for their preferred alternatives, they ran campaigns to influence public opinion and built alliances, for example, with the wider non-tobacco business community. Importantly, the study illustrates that TI strategies need to be seen and are tailored to specific contexts, in this case, among others, sensitivities around race and the legacy of Apartheid. It further shows TI’s efforts to redirect attention from the proposed regulation to TC areas (i.e. illicit trade and taxation) governed by other actors and processes, likely more favourable to TI preferences.

Findings on TI strategies resonate with literature on TI influence and other industries in SA. They are consistent with Zatoński et al.’s (2021) media-based work highlighting the use of economic arguments in TC policy-making. In line with Ulucanlar et al.’s (2023) identification of similar corporate political activities across unhealthy commodity industries, there is also a striking resemblance between the strategies identified here and those previously identified for other industries in SA. For example, the alcohol industry funded research to influence the Control of Marketing of Alcoholic Beverages Bill, which aimed to protect the public, especially children from a widely available, addictive unhealthy commodity (Bertscher et al. 2018). TI action- and argument-based strategies identified also closely align with those in other countries (Ulucanlar et al. 2016), including LMICs (Egbe et al. 2019, Bhatta et al. 2020, Matthes et al. 2021). There were notable parallels between JTI’s #HandsOffMyChoice campaign in SA and the #HandsOffMyPacks campaign, which opposed the planned standardised packaging policy in the UK and was run by an industry-funded smoker’s rights group (Tobaccotactics 2020a). The findings also illustrate industry efforts to portray illicit tobacco trade as caused solely by other supply chain actors, which has previously been identified as a key component of the industry’s global long-term influence strategies (Hird et al. 2022).

This study underlines the importance of contextual issues, most prominently, SA’s colonial history, the legacy of Apartheid, and persistent social, economic, and political challenges (Clark and Worger 2022). These issues are particularly apparent in two ways. First, the TI is heterogeneous, as seen in the rift between TISA and FITA. According to some participants, there may be a public perception that TISA represents the entrenched dominance of transnational corporations, predominantly staffed and controlled by wealthy white foreigners, while FITA represents local producers and transformation towards an economically empowered black population. More recently, it appears that transnational corporations seek to appear BEE compliant and improve their image by supporting black farmers, as seen with SATTA’s establishment.

Secondly, corruption is a major issue within SA, impacting industry interference and TC. Using campaigns, the TI alluded to corruption and illicit trade, as part of a strategy to oppose tax increases and standardized packaging. Article 5.3 of the WHO FCTC aims to minimize TI access to public health policymakers; corruption may make it harder to implement (Gilmore et al. 2015, Tobaccotactics 2024a). In this study, corruption added further challenges to addressing the illicit tobacco trade, where TISA used state power to spy on FITA. Conversely, FITA also has links to political elites, which could enable its TI members to conduct illicit activities without fearing the consequences.

This study brought together three conceptual frameworks, including two approaches to policy formulation in LMICs. We recommend researchers consider combining these frameworks in other case studies or integrate them into a single, concise, framework. The HPT enabled us to capture key contextual factors separate from the policy process, the PDM allowed us to identify TI strategies, and the BitM helped to capture these strategies within the policy formulation process.

The study has limitations. Given that it is mostly interview based, it is likely to not fully capture industry strategies. Gaps in participants’ accounts—conscious or not—cannot be ruled out (Diefenbach 2009). Some industry activities, such as informal interactions, might be less likely to be reported or be based on suspicions. To address this, we talked to a wide range of stakeholders, including two industry representatives and civil servants, and sought to triangulate interview data with other material. However, given the hidden nature of some forms of TI interference, not all claims by participants could be triangulated. Another limitation is that despite aiming to explore industry interference in TC more broadly, the focus was on the draft Bill. We therefore did not capture other policy areas (taxation and illicit trade) in detail. Our focus on industry interference also meant that we did not explore advocacy activity in relation to the draft Bill and TI interference. Future work could use a recent evidence-based classification of advocacy counterstrategies (Matthes et al. 2023) as a starting point. A final limitation was that our study did not capture how the COVID-19 pandemic impacted the policy formulation process for the draft Bill as our interviews were conducted before the outbreak. Future research could seek to understand how such emergency events shape policy formulation in LMICs.

Although beyond the period covered in this case study, it is important to note that after further delays, partially linked to the COVID-19 pandemic, the draft Bill was introduced in parliament in 2022. This lead TC advocates to hope that the draft Bill would become law in 2023 (Protect Our Next 2023). While public hearings were held in most of SA’s provinces in 2023 and 2024 (Portfolio Committee On Health 2024), as of early 2025, the draft Bill had not yet passed. Future work could study more recent TI activities.

This paper underlines the need for the SA government to fully implement the WHO FCTC and the WHO Protocol to Eliminate Illicit Trade in Tobacco Products in order to further strengthen TC in the country. Given the instances of TI interference identified here, compliance with Article 5.3 are essential and particularly pressing outside the health sector where such engagement tends to be more limited (Ralston et al. 2022). Broader government cooperation may also help with addressing the country’s illicit tobacco trade, as the implementation of the WHO Protocol will require extensive input from non-health bodies.

Effective implementation of Article 5.3 should go hand in hand with efforts to address corruption, identified as a key avenue for TI influence, as well as an important factor for illicit tobacco trade (Gallien 2020). SA is currently ranked 72 out of 180 countries featured in the Corruption Perceptions Index (Transparency International n.d.), and so efforts to improve this score would help facilitate more effective Article 5.3 implementation. The involvement of local researchers, advocates, and the public in monitoring and exposing the activities of the TI—and its attempts at state capture—is critical to preventing and countering TI interference and should be supported by the global TC community.

## Data Availability

No data are available.

## Reflexivity statement

The team of authors includes a mix of genders (one woman and three men), geographies (Germany, Poland, Scotland, and South Africa), and areas of expertise. One author is a South African (A.B.) national with expertise in alcohol industry interference in policy-making in South Africa and the tactics of unhealthy commodities more broadly. Another author (M.Z.) led this fieldwork and previously worked on TI interference in South Africa and had ample experience in researching TI interference in several countries, especially in Eastern Europe. The other two authors (A.W.A.G. and B.K.M.) have previously worked on TI interference in low- and middle-income countries.
